# Deciphering the formation of biogenic nanoparticles and their protein corona: State-of-the-art and analytical challenges

**DOI:** 10.1007/s00216-025-06144-z

**Published:** 2025-10-12

**Authors:** Andrés Suárez Priede, Miguel Gómez-Sánchez, Paula García-Cancela, Jörg Bettmer, Paula Díez

**Affiliations:** 1 Department of Physical and Analytical Chemistry, Faculty of Chemistry, University of Oviedo, Oviedo, Spain; 2 Health Research Institute of the Principality of Asturias (ISPA), Oviedo, Spain; 3 Department of Functional Biology (Immunology Area), Faculty of Medicine and Health Sciences, University of Oviedo, Oviedo, Spain

**Keywords:** Metallic nanoparticles, Green synthesis of nanoparticles, Biogenic nanoparticles, Bioanalytical characterization techniques, Protein corona

## Abstract

Biogenic synthesis of nanoparticles (NPs) using biological organisms such as plants, fungi, and bacteria offers a sustainable, green alternative to conventional chemical synthesis. These green methods involve biological matrices mediating metal ion reduction to form NPs capped by complex biomolecular coronas. These protein coronas critically affect NP stability, biological identity, and functionality, including notable therapeutic potentials. Despite advancements, the dynamic and heterogeneous nature of in situ biogenic NP formation remains insufficiently understood, as most studies emphasize synthetic production using biological extracts combined with metal precursors. Moreover, characterization of biogenic NPs requires a suite of bioanalytical techniques, each offering diverse advantages but also inherent limitations, requiring a comprehensive understanding of their features to critically select the optimal combination of strategies to obtain a full descriptive picture of the studied NPs. This review critically discusses current biogenic synthesis methods alongside the complementary bioanalytical strategies employed to elucidate NP formation, corona composition, and their biological implications. By highlighting the strengths and weaknesses of available techniques, the review aims to identify key challenges and future directions necessary to advance the safe and effective development of green nanotechnologies.

**Graphical Abstract:**

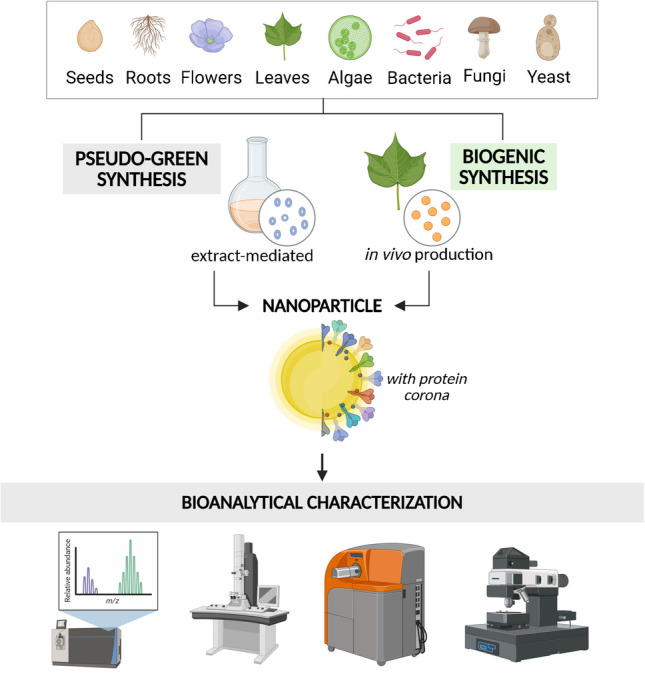

## Introduction

Nanomaterials (NMs), typically defined as objects with dimensions between 1 and 100 nm, exhibit various electronic, optical, and catalytic properties that have promoted their integration in diverse fields such as the chemical industry, pharmacy, medicine, and food science in this century [1]. The chemical diversity of these materials is vast, encompassing purely organic, (semi-)metallic, and composite structures, as well as quantum dots. Beyond chemical composition, parameters such as size, shape, crystal structure, aggregation state, and surface modification are critical determinants of their functional applicability. To exert precise control over these characteristics, a variety of synthetic strategies have been developed. Conventional approaches include bottom-up (chemical) and top-down (physicochemical) methods, both of which enable the fabrication of NMs with tailored properties, though often at the cost of using hazardous chemicals, solvents, or requiring sophisticated instrumentation [2]. In response to these limitations, alternative “green” synthesis concepts have emerged, leveraging enzyme-mediated processes, plants or fungal extracts, or even the biosynthetic capabilities of living microorganisms. These strategies generally follow the “traditional” bottom-up principles, wherein biological matrices are exposed to precursor ions to yield nanostructures with unique properties. The spectrum of NMs produced via such green methods is broad, including metals and semimetals such as Ag, Au, Cu, Zn, Fe, and Se, among others [3]. However, the main challenges remain the controllable and reproducible synthesis of NMs, especially regarding core composition, particle dimensions, and surface modifications. These factors are decisive, as biogenic NMs have evidenced promising properties for medical and pharmaceutical applications, including antimicrobial, anti-inflammatory, and anti-cancer activities, which continue to be the focus of extensive research efforts.

The complexity of biological reaction media, compared to controlled chemical systems, leads to NMs with greater variability and structural diversity. For instance, the size and shape of biogenic nanoparticles (bioNPs) produced by living organisms are influenced by the uptake of precursor materials and the intricacies of intracellular reduction and conversion processes. Such complexity requires advanced molecular-level characterization. While traditional analytical techniques, such as electron microscopy and X-ray spectrometry, provide valuable insights into particle cores, comprehensive characterization of biogenic NMs requires more sophisticated bioanalytical tools. This need arises because, unlike chemically synthesized NPs, where reducing and capping agents are well-defined, bioNPs are stabilized by a diverse array of biological molecules, the nature of which depends on the synthetic pathway and organism used.

A particularly important aspect in the study of NPs is the formation of the protein corona (PC), a dynamic layer of biomolecules that adsorbs onto the NP surface upon exposure to biological environments [4]. The PC critically affects NP identity, behaviour, and biological interactions, thereby impacting their efficacy and safety in biomedical applications. Consequently, the development and application of robust bioanalytical strategies are essential for elucidating the composition, dynamics, and functional implications of the PC.

This review aims to critically assess recent advances in bioanalytical methodologies for the characterization of bioNPs, with a special emphasis on strategies for probing PC formation. We will discuss the challenges and opportunities associated with current analytical approaches, highlight their relevance for understanding NP-biomolecule interactions, and outline future directions for the field.

## Diversity of biogenic nanoparticle production: Living organism-based pathways and artificial extract approaches

**Fig. 1:**
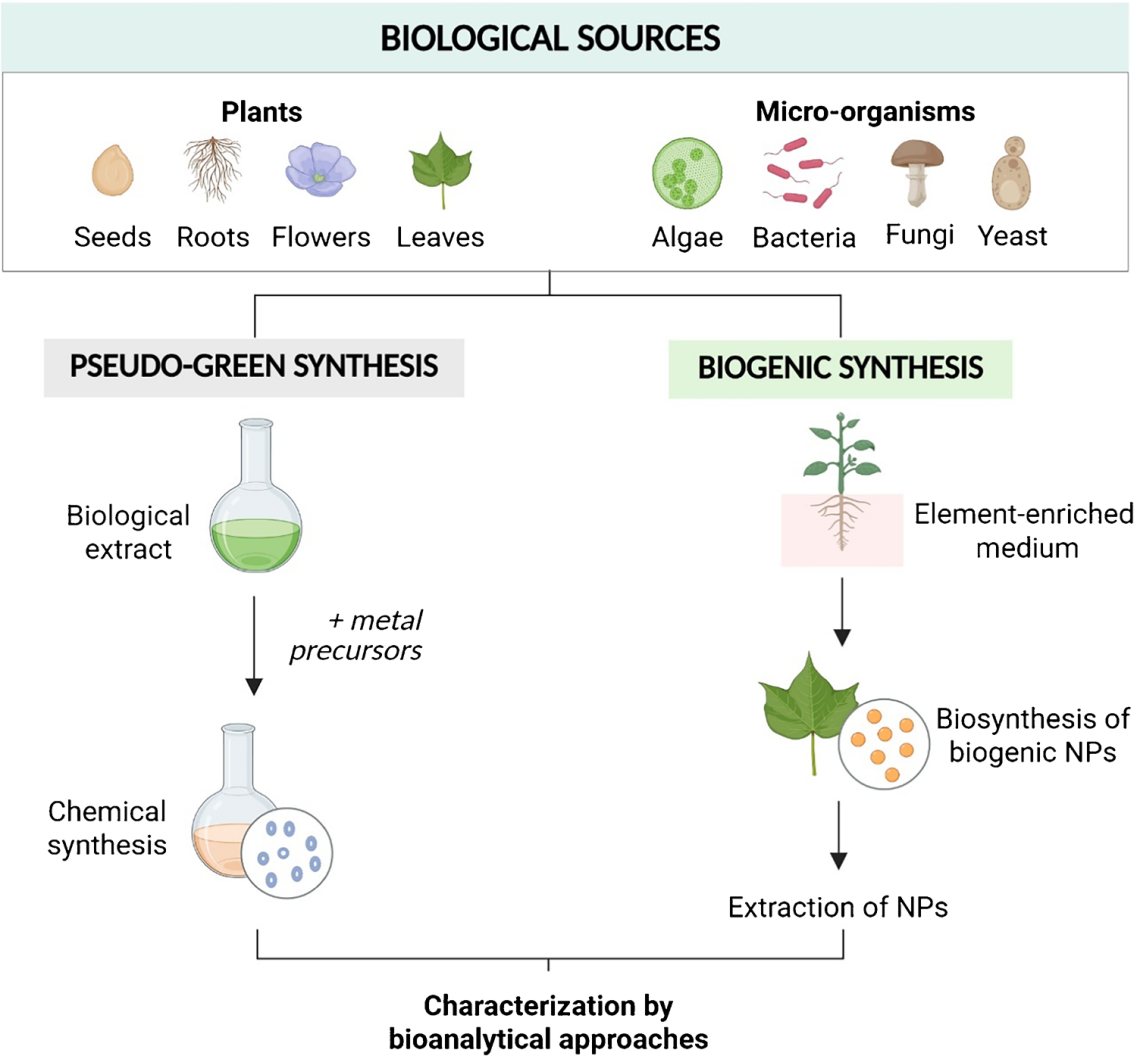
Summary of biological sources and methodologies for the synthesis of nanoparticles with biological origin. Two main synthesis strategies are depicted: pseudo-green synthesis, where biological extracts are combined with metal precursors to chemically synthesized NPs, and biogenic synthesis, where living organisms are cultured in element-enriched media, leading to the in vivo biosynthesis of biogenic nanoparticles, which are subsequently extracted. *NPs*, nanoparticles. [Figure created with BioRender.com]

In recent years, biogenic synthesis has emerged as a promising and environmentally friendly approach for the fabrication of NPs. Unlike conventional physical and chemical methods, which often involve toxic reagents and high energy consumption, biogenic synthesis employs biological systems to produce NPs under mild conditions (Fig. 1). This green chemistry route not only reduces environmental impact but also offers better biocompatibility and scalability [5]. The unique metabolites and biomolecules produced by these organisms act as natural reducing and stabilizing agents, facilitating the formation of NPs with a wide range of morphologies and physicochemical characteristics.

**Fig. 2:**
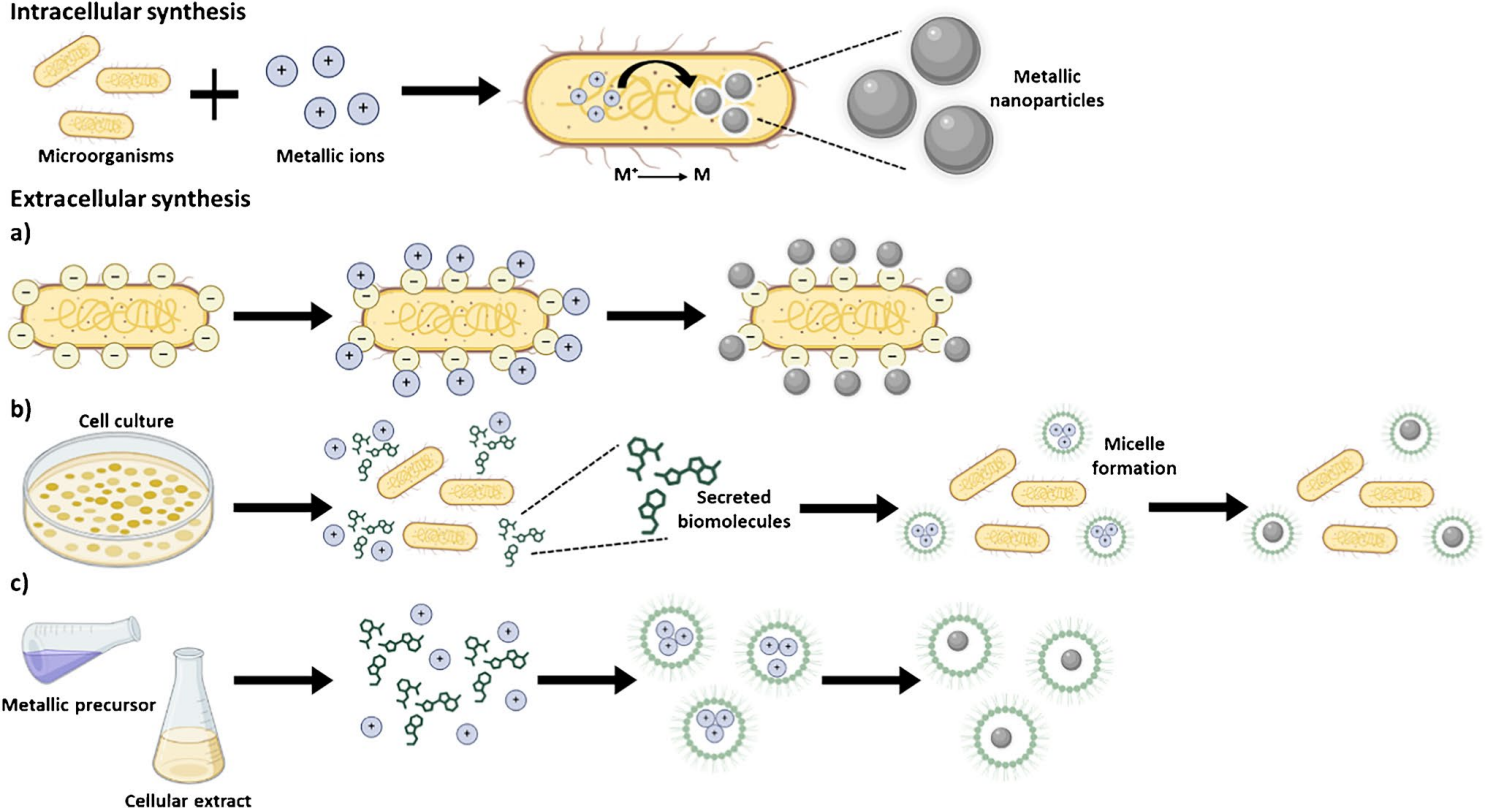
Mechanisms of microorganism-mediated synthesis of nanoparticles. Among the extracellular mechanisms: (a) synthesis in which the microorganism itself acts as a “template” for nucleation, (b) synthesis in which biomolecules excreted by the microorganisms induce nucleation, and (c) synthesis in which biomolecules present in the cell extract induce nucleation. [Figure created with BioRender.com]

Green biogenic synthesis follows bottom-up approaches, as it relies on the assembly of particles from molecular or atomic units [6]. Although the exact mechanism of bioNP formation depends on the producer organism and the nature of the NP—and in many cases it is still not fully characterized—two main routes are generally distinguished: intracellular or extracellular synthesis (Fig. 2). Intracellular synthesis occurs inside the cells or tissues of the organisms, either in the cellular wall, the cytoplasm, or even inside organelles. Typically, positively charged metal ions interact with the negative charges of the cell wall, and they become internalized. Once inside, enzymatic systems, reducing metabolites such as NADH-dependent reductases, and other cellular biomolecules mediate the reduction of metal ions to their zero-valent form, which subsequently nucleate and grow into nanoparticles [7]. This process usually produces more stable particles with controllable size and shape, although subsequent cell lysis and complex purification protocols are required [8]. In extracellular synthesis, free metal ions aggregate onto the surface of the cells and are then reduced by enzymes from the cell wall or by secreted biomolecules in the culture medium, such as enzymes, proteins, polysaccharides, or phenolic compounds. These compounds act as both reducing and capping agents, converting metal ions present in the medium into stable nanoparticles and preventing uncontrolled aggregation [9]. The final size, shape, and surface chemistry of the resulting bioNPs will depend on the type and concentration of biomolecules present, as well as environmental factors such as pH, temperature, and metal ion concentration [7]. This approach facilitates easier recovery of NPs and is generally more scalable [10].

Despite the numerous advantages of bioNP synthesis, several intrinsic challenges remain. A key issue is the batch-to-batch variability, which arises from variations in organism physiology, growth conditions, and metabolite profiles. This biological heterogeneity affects reproducibility and complicates the standardization of NP size, shape, and functional properties. Furthermore, the complex biological matrices involved often hinder downstream purification, requiring additional steps to effectively remove residual biomolecules, cell debris, and unreacted precursors. While extracellular synthesis routes offer improved scalability relative to intracellular methods, large-scale production of bioNPs still faces critical limitations. These include difficulties in exerting precise control over particle size distribution, shape uniformity, and inter-batch reproducibility [11]. Such limitations contrast with approaches using purified biomolecules like peptides, proteins, or nucleic acids [12, 13], which allow highly controlled and reproducible NP structures through defined biochemical interactions and controlled reaction conditions.

Nevertheless, biogenic methods are generally simpler and more cost-efficient to scale up since they harness the intrinsic biochemical diversity and natural reducing/stabilizing agents present in living organisms, without the need for labor-intensive purification of biomolecules. This simplicity translates into an attractive green chemistry alternative with lower environmental impact and resource requirements. Addressing current challenges while maximizing these distinctive advantages is essential to unlock the full potential of bioNPs. Improved process control, standardization protocols, and integrated scale-up strategies will be critical to advance bioNPs toward widespread applications in biomedicine, environmental remediation, and beyond.

### Living systems as nanofactories of bioNPs

**Fig. 3:**
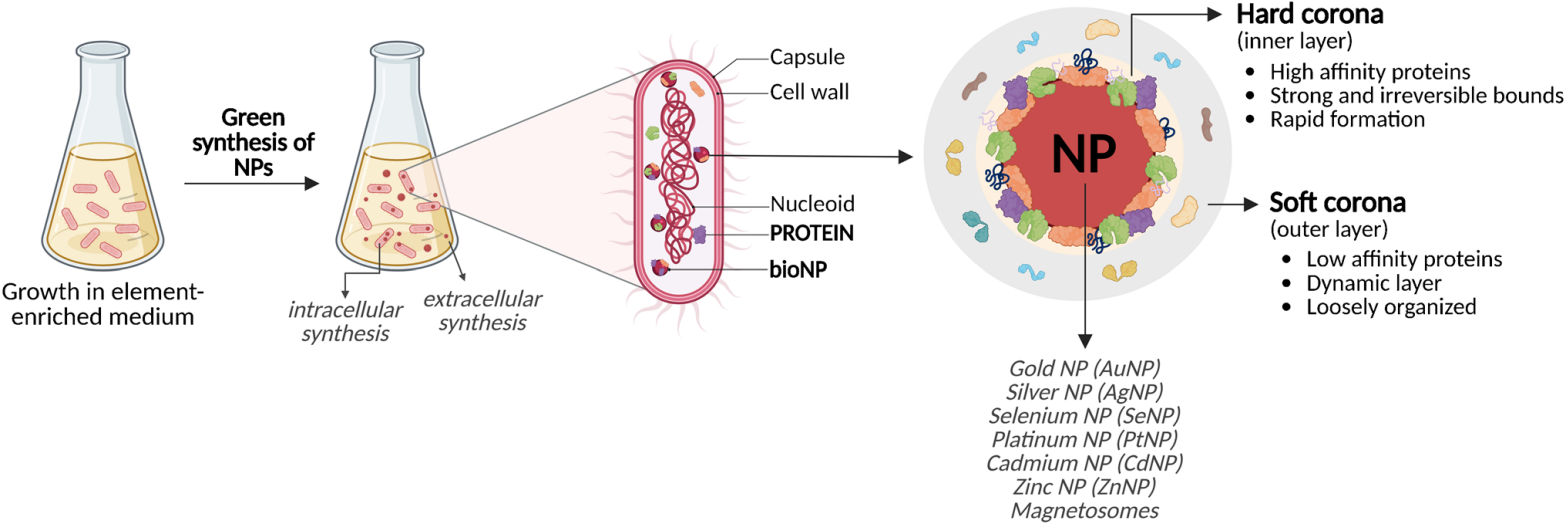
Schematic representation of the green synthesis of biogenic nanoparticles (bioNPs) using bacteria as an example of a biological source. bioNPs are produced intra- or extra-cellularly and are accompanied by a protein corona composed of two distinct layers: an inner “hard corona” and an outer “soft corona”. *NPs*, nanoparticles; *bioNP*, biogenic nanoparticle. [Figure created with BioRender.com]

A wide variety of living organisms have been reported to mediate the biogenic synthesis of NPs (Figs. 1 and 3). These include microorganisms such as bacteria, fungi, and algae, as well as numerous plant species [14].

Bacteria are abundantly present in the environment, can be cultivated easily, and can adapt to different environmental conditions such as temperature, pressure, and pH. This adaptability makes them good candidates for NP synthesis, as changes in these conditions play a key role in controlling the amount and size of the NPs produced [15]. Bacteria are capable of both intracellular and extracellular NP synthesis, often associated with microbial resistance processes and cellular detoxification systems mediated by specific enzymes [16]. However, bacterial synthesis of nanostructures presents certain limitations, particularly related to purification protocols and the difficulty in controlling the shape and size of the resulting NPs [14]. Many bacterial species have been reported to produce silver NPs (AgNPs), including *Lactobacillus acidophilus*,* Bacillus cereus*, *Arthrobacter gangotriensis*, *Pseudomonas proteolyticus*, *Escherichia coli*, and *Aggregatimonas sangjinii* [6, 17]. Other species, such as *Streptomyces*, *Rhodococcus*, *Nocardia*, *Lactobacillus*, and* Staphylococcus* [14] have been employed in the synthesis of gold NPs (AuNPs). Also, stable colloidal dispersions of AuNPs were spontaneously formed extracellularly when *Cupriavidus metallidurans* was exposed to Au solution, as reported by Montero-Silva et al*.* [18]. Bacteria can also produce metal oxide NPs like ZnO [19] or iron oxide magnetic nanostructures such as magnetosomes [20]. The synthesis of quantum dots like CdSe nanostructures has also been carried out by means of bacteria like *Staphylococcus aureus* [21]*.*

Fungi are also well recognized for their ability to bioaccumulate metals and produce NPs. They contain enzymes that are particularly advantageous for biosynthesis compared to other microorganisms [22] and can follow both intracellular and extracellular synthesis routes. Intracellular synthesis mainly occurs in the mycelia [14], while extracellular generation is facilitated by the efficient secretion of proteins and enzymes, often resulting in higher stability of the final nanostructures [10]. Several fungal species, such as *Cladosporium cladosporioides* [23], *Aspergillus flavus* [24], *Penicillium* sp. [25], *Mariannaea* sp. [26], *Ganoderma Lingzhi* [27], *Phycomyces blakesleeanus* [28], and *Fusarium oxyporum* [29] have been described as producers of gold, silver, selenium, platinum, and cadmium NPs. Fungi can also synthesize metal oxide NPs, such as cobalt oxide [30] and magnetic iron oxide [31]. Likewise, yeasts, which are single-celled eukaryotic fungi, can also drive the reduction of metal ions into NPs, primarily through their abundant secretion of enzymes. Their rapid growth makes them easy to cultivate and preserve [22], and they typically yield larger quantities of NPs compared to bacteria [14]. Species such as *Candida guilliermondii* [32], *Candida utilis* [33], *Rhodotorula glutinis* [34], *Saccharomyces cerevisiae* [35, 36], *Schizosaccharomyces pombe*, and *Candida glabrata* [37] have been reported for the synthesis of gold, silver, selenium, cadmium, and zinc sulfide NPs, as well as quantum dots and semiconductor nanostructures.

Algae, both unicellular and multicellular [10], possess significant potential for NP biosynthesis due to their ability to accumulate and reduce metals, as well as their production of enzymes and pigments involved in bioreduction [14]. Although direct studies on algae-mediated NP synthesis are limited, some examples include the production of gold and silver NPs by *Tetraselmis kochinensis* [38], *Chromochloris zofingiensis* [39], and *Desmodesmus* sp. [40]. Most research, however, focuses on the use of pre-prepared algal extracts.

Plants represent an accessible and manageable resource for bioNP synthesis, offering advantages over microorganisms, particularly in terms of scalability [41]. The diverse range of phytochemicals present in plants—including flavonoids, amino acids, phenolic compounds, enzymes, alkaloids, polysaccharides, and tannins—plays key roles in the reduction of metallic ions [14], making plants excellent candidates for green NP synthesis [42]. While most studies focus on the use of plant extracts to avoid the complex processes of NP recovery from plant tissues, some research has documented intracellular NP formation in living plants of *Medicago sativa* (alfalfa) following ion uptake from the environment [43]. Also, *Brassica juncea* [44] and *Sesbania drummondii* [45] have been shown to form and accumulate gold and silver NPs internally.

### Extract-mediated “green” synthesis of NPs

In addition to direct biosynthesis by living organisms, there is a growing trend toward the use of “artificial” or pre-prepared extracts from plants, algae, or fungi for NP synthesis (Fig. 1). In these methods, biomolecules and metabolites are first extracted from the precursor organism using techniques such as heating, maceration, mechanical or enzymatic lysis, solvents, or sonication. These extracts are then used as reagents for the “green” synthesis of NPs [46]. While this approach is practical and scalable, the extraction protocols can modify the structures and properties of the biomolecules involved in reduction and stabilization, leading to differences in the properties of the resulting NPs and their PC compared to those produced directly by living organisms. Plant extracts are widely employed, with various parts of the plant—fruit, root, leaves, and bark—used to produce NPs of metals such as gold, silver, copper, and iron, as well as metal oxides [47]. Algae and fungi are also employed to this end [48, 49]. The reaction conditions, including temperature, ionic concentration, and reaction time, significantly influence NP size and yield [50].

In summary, biogenic synthesis represents a versatile and environmentally responsible route to NP production, leveraging the biochemical diversity of living systems. The choice of organism and synthesis route—intracellular, extracellular, or extract-mediated—greatly influences NP properties, scalability, and downstream applications. Understanding these biological processes is essential for optimizing NP synthesis and tailoring their physicochemical characteristics for specific uses, especially in biomedical contexts where the nature of the PC and surface chemistry are critical for function and safety.

### Applications of biogenic nanoparticles

Supported by their intrinsic biocompatibility, eco-friendly production routes, and the stabilizing role of biomolecules attached to their surfaces, bioNPs have attracted increasing attention across biomedical, environmental, agricultural, analytical, and industrial domains. The biomolecular corona conferred during their synthesis not only enhances colloidal stability but also endows bioNPs with additional bioactivity and functional selectivity.

In the biomedical field, numerous plant- and fungi-derived bioNPs have demonstrated potent antimicrobial [51], anti-cancer [52], and antioxidant activities [53]. Beyond their intrinsic therapeutic properties, bioNPs are being developed as nanocarriers for drug delivery [14], imaging probes, and as functional elements of biosensors designed for the detection of nucleic acids, proteins, metabolites, and even pathogenic microorganisms [54]. Recent studies also highlight their role in tissue engineering and regenerative medicine, where the combination of nanoscale features and biomolecule interactions promotes cell adhesion and differentiation.

In environmental applications, bioNPs synthesized from diverse organisms have emerged as effective and sustainable tools for pollution mediation. Fe- and Ag-based bioNPs have demonstrated strong catalytic activity, accelerating the degradation of organic dyes, pesticides, and other contaminants from the environment and wastewaters [55]. Their potential is being further investigated for the remediation of recalcitrant pollutants such as pharmaceuticals, personal care products, and microplastics, which are a rising global concern.

The agricultural and food sectors also benefit from the versatile functions of bioNPs. ZnO- and Ag-based bioNPs are increasingly applied as biofertilizers and growth-promoting agents, enhancing nutrient uptake and crop resilience under stress conditions. In food science, biogenic nanostructures are incorporated into food packaging systems as antimicrobial coatings to improve shelf life, as well as into nanosensors for the rapid detection of foodborne pathogens and toxins [15].

In analytical science, the surface biomolecules associated with bioNPs confer unique recognition and catalytic features that can be exploited in colorimetric, electrochemical, and SERS-based assays [54]. These properties enable highly sensitive and selective detection platforms with applications ranging from medical diagnostics to environmental monitoring. Their compatibility with microfluidic formats is accelerating the path toward point-of-care devices capable of onsite analysis. Only a few studies showed the applicability of differently synthesised bioNPs as (bio)sensors. One example is about the synthesis of Ag nanoparticles produced by a green algae extract that allowed the colorimetric determination of hydrogen peroxide with detection limits in the low nM range [56]. This application field might be a challenging topic to explore in the future.

Looking ahead, the integration of bioNPs into next-generation nanotherapeutics, sustainable agro-technologies, and environmental remediation platforms is likely to expand further. Their adaptability across distinct sectors, combined with the growing emphasis on green and circular production methods, positions biogenic nanotechnology as a key enabler of translational nanomedicine, sustainable chemistry, and global health solutions.

## Current approaches for extracting and isolating bioNPs

A deep analytical characterization of any bioNPs requires the careful and representative isolation of the objects. The suggested methodologies are clearly defined by the purpose of the study: Which information should be generated, e.g., characteristics about the core properties of the NPs or additionally about their surface composition (PC)? Therefore, depending on the synthetic methods applied, such procedures can be quite exhaustive and prone to the loss of qualitative and/or quantitative characteristics of the sought objects.

**Fig. 4:**
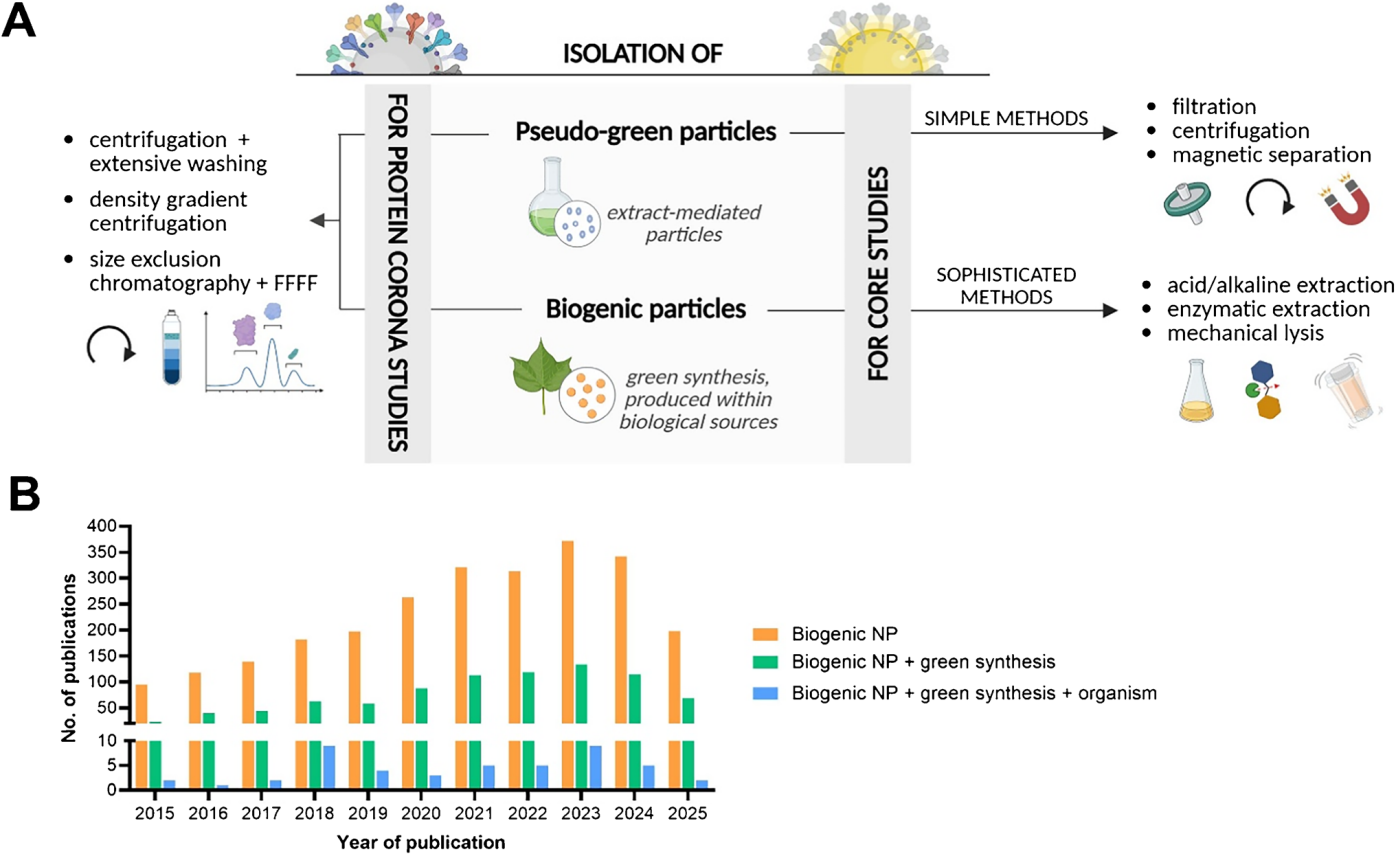
Strategies for the purification of nanoparticles synthesized from (pseudo-green particles) and within (biogenic particles) living organisms (such as plants, bacteria, yeast). **A** Illustration of different NP isolation approaches tailored to their intended application (i.e., core or protein corona studies). [Figure created with BioRender.com]. **B** Summary of PubMed-indexed publications (as of 28/07/2025) between 2015 and 2025, retrieved using the search terms “Biogenic NP” (in orange), “Biogenic NP + green synthesis” (in green), and “Biogenic NP + green synthesis + organism” (in blue)

The isolation of extract-mediated particles can utilize the most straightforward procedures (Fig. 4A). As the generated bioNPs are already suspended in a liquid phase, relatively simple analytical processes could isolate them. Besides filtration steps, centrifugation procedures followed by washing are among the most commonly applied methods to separate bioNPs from the matrix [57]. However, to the best of our knowledge, no systematic studies have been conducted to evaluate whether these steps affect the integrity of the bioNPs. In any case, the simplicity of these extraction or isolation methods might also be the driving force for the impressive number of studies using extract-mediated approaches for the synthesis of bioNPs (Fig. 4B).

The extraction of intracellular NMs from biological tissues, cells, or organisms requires different, more sophisticated strategies. Most methods used, at least demonstrated for engineered particles, refer to acidic or alkaline media as extraction media or are based on enzymatic approaches [58]. Relevant studies made use of the incubation of Ag- and/or Au-containing NPs of different sizes and surface modifications to prove their recovery and integrity after the applied extraction procedures. Incubations with tetramethylammonium hydroxide (TMAH) as an alkaline reagent showed to be efficient for the extraction of various biological tissues like organs from mice [59], ground meat [60] or spleen samples from Wistar rats [61]. In general, these approaches showed relatively high recovery of the administered NPs without the observation of significant degradation in terms of size. Enzymatically assisted extractions (e.g., using proteinase K) generally provided lower recoveries, as demonstrated in [61]. However, such an approach, in combination with H_2_O_2,_ showed applicability to more complex NP systems like CeO_2_ and TiO_2_ [62].

**Fig. 5:**
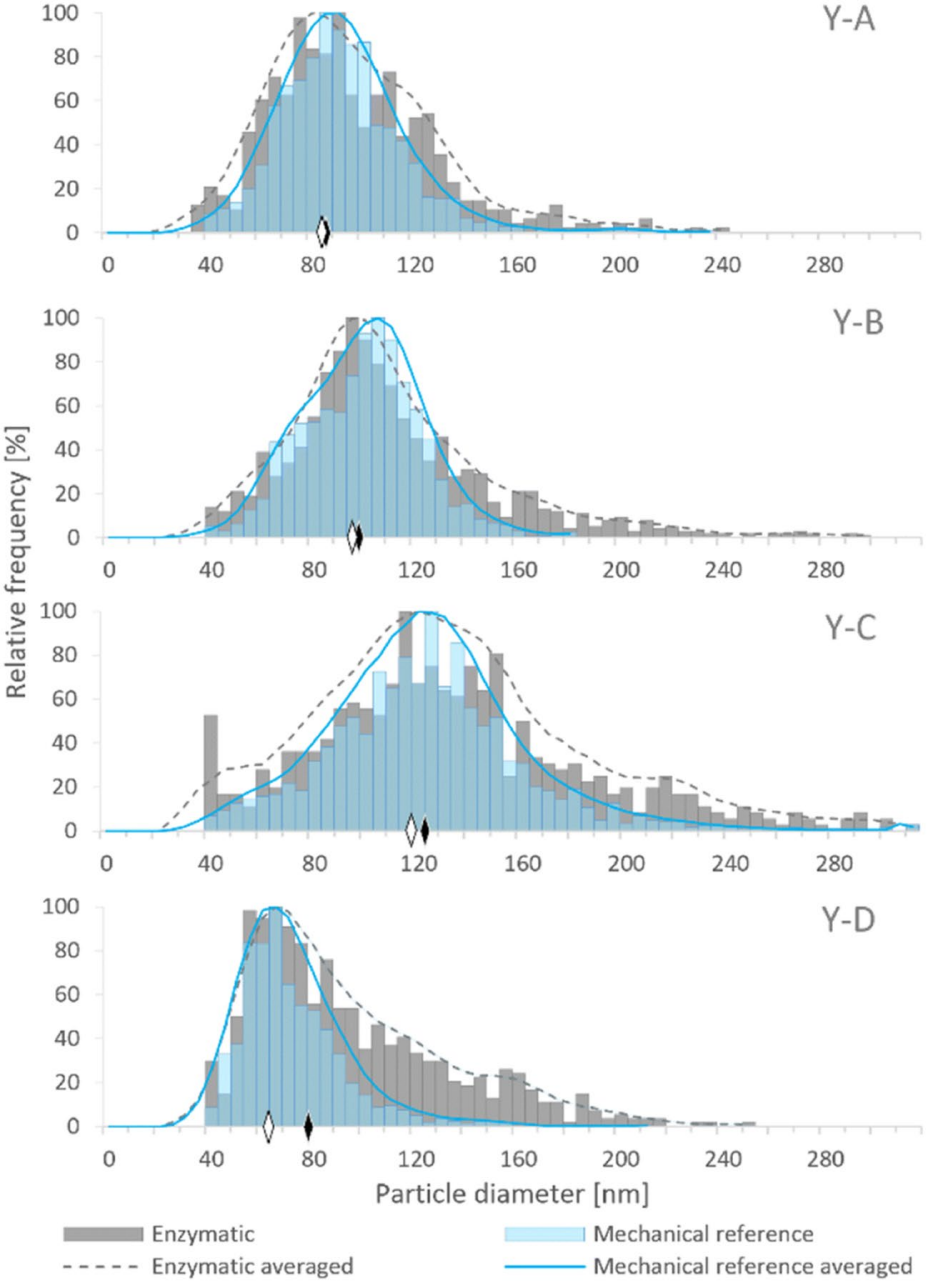
Histograms representing the SeNP size distribution in four Se-enriched yeast samples after enzymatic (grey columns) and mechanical (blue columns) cell lysis procedures. The solid and dashed lines represent the averaged values of the particle size distribution after the enzymatic and mechanical cell lysis procedures, respectively. The diamonds represent the median size of SeNPs after enzymatic (black) and mechanical (white) cell lysis procedures. Bin size: 5 nm [Reproduced from [64] with permission from The Royal Society of Chemistry]

Extraction and isolation of bioNPs were probably best studied for selenium-containing NMs. For instance, the synthesis of SeNPs in lactic acid bacteria was followed by an extraction that involved the use of sodium dodecyl sulphate (SDS) and NaOH [63]. An enzymatic approach showed the possibility of isolation of biogenic SeNPs from yeast (*Saccharomyces cerevisiae*) enriched in selenium [35]. A purely mechanical disruption and lysis of yeast cells and the mycelium of *Ganoderma lingzhi* made use of glass beads in combination with vortex and ultrasonic treatment, capable of releasing biogenic SeNPs from the biological matrix [27, 36]. A systematic study on a total of ten different extraction procedures, including chemical, enzymatic, and mechanical approaches, revealed a significant influence on the results [64]. It could be observed that the procedures generally affected the amount of extracted SeNPs, their size distribution (Fig. 5), and the dissolution and aggregation of SeNPs. These results confirmed that extraction strategies might artificially influence the nature of the originally present bioNPs and reflect somehow the challenges to overcome for a reliable characterization of these species.

The above-mentioned methods might provide a relevant characterisation of the particle core properties. The use of enzymes or reagents like SDS, bases, or acids impedes their applicability for the analysis of the PC of engineered NPs or even bioNPs [65]. Thus, other “softer” strategies were developed for the isolation of the entire NP-PC complexes, basically tested for biological fluids incubated with engineered particles. Among them, centrifugation in combination with extensive washing is the most common one [66]. To increase the separation efficiency between the sought complexes, the matrix, and other dissolved proteins, density gradient centrifugation (DGC) showed promising results, at least without changing the composition of the PC [67]. First reports appeared around the analysis of the PC formed in human cells after the incorporation of Au NPs using DGC. For this, a soft cell lysis initiated the release of the NP-PC complex from MCF-7 [68], HCT-116, and A549 cells [69] with subsequent DGC. Other more sophisticated techniques involved size-exclusion chromatography and flow field-flow fractionation, but they were generally accompanied by important disadvantages, as summarized recently [65]. Regarding the fact that basically only biological fluids were investigated, there is still plenty of room for improvement and systematic studies for suitable and reliable sample preparation protocols. To our best knowledge, bioNPs have never been exhaustively studied for the composition of the PC after appropriate sample preparation.

In general, existing extraction strategies are fit-for-purpose, but more studies are needed to evaluate their potential, pitfalls, and drawbacks for bioNPs. Especially, the isolation of the PC (at least the hard PC) from biological sources requires extensive investigations in order to further understand the biogenic NP formation and their potential applications.

## Core characterization techniques for green-synthesized nanoparticles

**Fig. 6:**
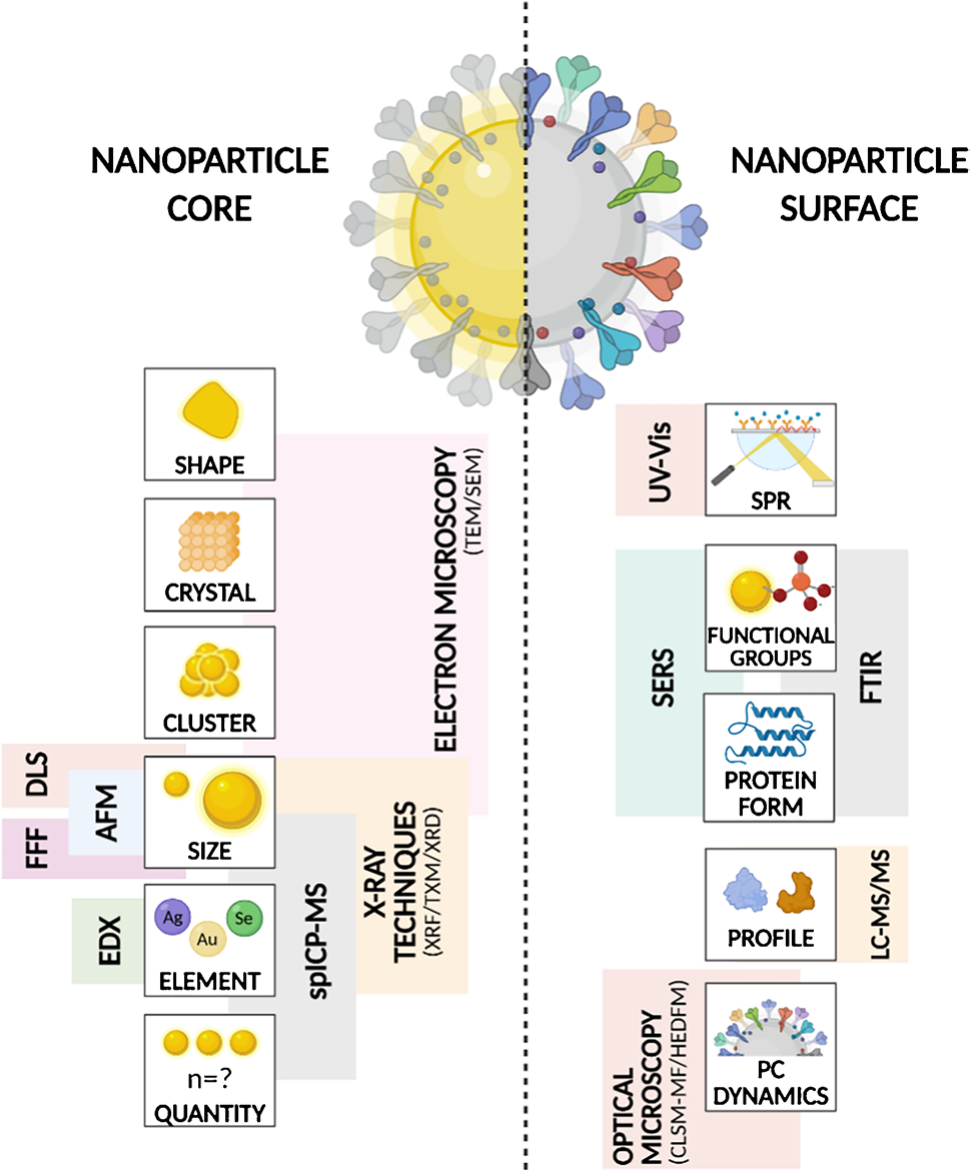
Overview of bioanalytical characterization techniques applied to nanoparticle core and surface properties. *AFM*, atomic force microscopy; *CLSM-MF*, confocal laser scanning microscopy with microfluidics; *DLS*, dynamic light scattering; *EDX*, energy-dispersive X-ray spectroscopy; *FFF*, flow field fractionation; *FTIR*, Fourier transform infrared spectroscopy; *HEDFM*, hyperspectral-enhanced dark-field microscopy; *ICP-MS*, inductively coupled plasma mass spectrometry; *LC-MS/MS*, liquid chromatography tandem mass spectrometry; *PC*, protein corona; *SEM*, scanning electron microscopy; *SERS*, surface-enhanced Raman spectroscopy; *SPR*, surface plasmon resonance; *TEM*, transmission electron microscopy*; TXM*, transmission X-ray microscopy*; UV-Vis*, ultraviolet-visible spectroscopy; *XRD*, X-ray diffraction; *XRF*,* X-ray fluorescence.*[Figure created with BioRender.com]

As with synthetic NPs, thorough characterization of core properties—size, shape, and composition—is essential, as these properties dictate their physicochemical behaviour and potential uses. To this end, various bioanalytical techniques can be applied (Fig. 6, Tables 1 and 2).

**Table 1:** Summary of main features of bioanalytical strategies for characterizing the nanoparticle cores

Bioanalytical strategy	Technique	Evaluated parameters											Advantages	Limitations	Cost	Processing time
		Size	Shape	Crystallinity	Surface morph	Aggregation	3D imaging	Hydrodyn. diam	Zeta potential	Elemental comp	Concentration	Optical features				
Electron microscopy	TEM	x	x	x									High-resolution, atomic detail	Extensive sample preparation, vacuum, possible artefacts, low throughput	High	Hours
	SEM	x	x		x	x							High-resolution, easier preparation (vs TEM)	Lower resolution (vs TEM), conductive coating needed	High	Hours
Atomic force microscopy	AFM	x					x						Versatile (air/liquid), minimal preparation, high-resolution surface detail	Low throughput, tip-sample artefacts	Moderate–high	Minutes to hours
Dynamic light scattering	DLS	x						x	x				Rapid, non-destructive	Poor performance on polydisperse/non-spherical samples	Low–moderate	Minutes
X-ray-based techniques	XRD	x		x									Non-destructive	Needs crystalline material	Moderate	Minutes to hours
	XRF/TXM						x			x			Elemental mapping, in situ, 3D possible	Shallow penetration, semi-quantitative	High	Minutes to hours
Energy-Dispersive X-ray Spectr.	EDX									x			Fast, micro/nanoscale composition	Shallow penetration, semi-quantitative	Moderate–high	Minutes to hours
ICP-MS-based techniques	spICP-MS	x								x	x		Highly sensitive, quantitative, and works with complex matrices	Calibration required, assumes spheres	High	Minutes

*TEM*, transmission electron microscopy; *SEM*, scanning electron microscopy; *AFM*, atomic force microscopy; *DLS*, dynamic light scattering; *XRD*, X-ray diffraction; *XRF*, X-ray fluorescence; *TXM*, transmission X-ray microscopy; *EDX*, energy-dispersive X-ray spectroscopy; *spICP-MS*, single-particle inductively coupled plasma mass spectrometry

**Table 2:** Overview of studies characterizing biogenic nanoparticles using various analytical techniques. Both biogenic nanoparticles—synthesized from biological sources such as bacteria, plants, fungi, yeast, or seaweed—and pseudo-green synthesis approaches are included

Technique	Type of NP	Biological source	Studied feature	Outcome	Reference
TEM	AuNPs	Bacteria (*S. epidermidis*)	Size, shape, agglomeration	20–25 nm	[71]
	ZnONPs	Plant (*B. tomentosa*)	Size, shape, agglomeration	22–94 nm	[72]
	CuNPs	Plant (*C. arnotiana*)	Size, shape, agglomeration	60–90 nm	[73]
SEM	Cu NPs	Plant (*M. oleífera*)	Size, shape, topography	5–20 nm	[74]
	CuO NPs	Plant (*A. altissima*)	Size, shape, topography	36–49 nm	[75]
	ZnO NPs	Plant (*A. lebbeck*)	Size, shape, topography	66–113 nm	[76]
AFM	AgNPs	Plant (*T. terrestris*)	Size, morphology	60 nm	[78]
		Fungi (*Aspergillus*)	Size, morphology	Spherical	[79]
		Bacteria (*Bacillus*)	Size, morphology	12–20 nm	[80]
DLS	AgNPs	Plant (*E. platyloba*)	Hydrodynamic diameter, Z potential	24 nm; −12 mV	[82]
		Plant (*A. diversifolia*)	Hydrodynamic diameter, Z potential	34 nm; −20.2 mV	[83]
		Plant (*M. azedarach*)	Hydrodynamic diameter, Z potential	32 nm; −13.1 mV	[84]
	AuNPs	Plant (*E. platyloba*)	Hydrodynamic diameter, Z potential	139 nm; −48 mV	[82]
XRF/TXM	AuNPs	Yeast (*S. cerevisiae*)	Composition, 3D imaging	NP presence inside cells	[85]
XRD	SeNPs	Bacteria (*P. aeruginosa*)	Structure, composition	Face-centered cubic structure	[86]
	AgNPs	Fungi (*R. solani*)	Structure, composition	High-purity crystalline structure	[87]
		Fungi (*C. cladosporioides*)	Structure, composition	High-purity crystalline structure	[87]
EDX	ZnONPs	Seaweed (*C. peltata*, *H. Valencia*,* S. myriocystum*)	Composition	52% Zn, 42% O	[88]
	FeONPs	Plant (*E. crassipes*)	Composition	77.08% Fe, 22.97% O	[89]
	Ag/Au NPs	Plant (*C. monogyna*)	Composition	Pure Ag and Au	[90]
spICP-MS	TeNPs	Bacteria (*S. aureus*,* E. coli*)	Composition, size, concentration	11–12 nm; Te presence	[92]
	CuNPs	Bacteria (*S. coelicolor*)	Composition, size, concentration	8–95 nm; Cu presence	[93]
	PoliP NPs	Bacteria (*S. coelicolor*)	Composition, size, concentration	P presence	[122]
AF4-ICP-MS	SeNPs	Bacteria (Lactic acid)	Composition, size, aggregation	247 nm; Se presence	[63]
UV/vis	AgNPs	Fungi (*M. phaseolina*)	PC formation	SPR peak displacement (414 to 420 nm)	[103]
	AuNPs (citrate-coated)	Synthetic	PC formation (BSA adsorption)	SPR peak displacement (534 to 537 nm)	[104]
	AgNPs (L-Met-coated)	Synthetic	PC formation (BSA adsorption)	SPR peak displacement (424 to 427 nm)	[105]
FTIR	SeNPs	Bacteria (*A. brasilense*)	Surface characterization	Proteins, carboxypolysaccharides, lipids	[107]
	AgNPs	Fungi (*N.oryzae*)	Surface characterization	Proteins	[108]
	AgNPs (different coatings)	Synthetic	Conformational changes in adsorbed proteins	Transitions from α-helices to β-sheet structures (amide I band shifts)	[109]
SERS	AuNPs	Synthetic	Fragmentation of PC inside cells	PC fragmentation upon cellular processing	[112]
LC–MS/MS	AgNPs	Fungi (*M. phaseolina*)	PC protein identification	46 proteins from the fungal proteome	[103]
	SeNPs	Yeast (*S. boulardii*)	PC protein identification	PEP4p as predominant protein	[115]
	AuNPs	Synthetic	PC protein identification	200 proteins	[116]
CLSM-MF	SiMPs	Synthetic	Real-time PC adsorption monitoring	Three different adsorption regimes	[117]
HEDFM-HIS	AgNPs	Synthetic	PC formation	Wavelength shifts in the scattered light	[121]

*TEM*, transmission electron microscopy; *SEM*, scanning electron microscopy; *AFM*, atomic force microscopy; *DLS*, dynamic light scattering; *XRF/TXM*, X-ray fluorescence/transmission X-ray microscopy; *XRD*, X-ray diffraction; *EDX*, energy-dispersive X-ray spectroscopy; *spICP-MS*, single-particle inductively coupled plasma mass spectrometry; *UV/Vis*, ultraviolet–visible spectrophotometry; *FTIR*, Fourier transform infrared spectroscopy; *SERS*, surface-enhanced Raman spectroscopy; *LC-MS/MS*, liquid chromatography coupled with tandem mass spectrometry; *CLSM-MF*, confocal laser scanning microscopy–multiphoton fluorescence; *HEDFM-HIS*, hyperspectral-enhanced dark-field microscopy–hyperspectral imaging spectroscopy; *NP*, nanoparticle; *PC*, protein corona

### Electron microscopy

Electron microscopy, including transmission (TEM) and scanning (SEM) electron microscopy, is fundamental for NP characterization due to its ability to provide information on particles’ size, shape, and agglomeration state. TEM, operating at 80–200 keV, can image NPs as small as 2 nm and reveal their morphology. The resolution of this equipment can be further improved using high-resolution TEM (HR-TEM), allowing for a spatial resolution of down to 0.047 nm and the determination of crystalline structures [70]. For example, AuNPs synthesised from *Staphylococcus epidermidis* [71], ZnONPs from *Bauhinia tomentosa* [72], or CuNPs from *Cissus arnotiana* [73] were characterized using TEM, offering information about shape and agglomeration state and the size ranges: 20–25 nm, 22–94 nm, and 60–90 nm, respectively. SEM, using lower voltages, provides detailed surface topography, as demonstrated in the analysis of Cu [74] and CuO [75] NPs from *Moringa oleifera* and *Ailanthus altissima* leaf extracts with 5–20 nm and 36–49 nm, respectively, or in the case of ZnO NPs from *Albizia lebbeck* stem bark, which ranged from 66 to 113 nm in various shapes [76]. In both cases, they are usually coupled to an EDX instrument to complement the information provided in terms of composition. The main disadvantages of these techniques in bioNPs analysis are the low throughput and the need for samples to be conductive in the case of SEM.

### Atomic force microscopy (AFM)

This technique offers near-atomic resolution for the analysis of particles as small as 9 nm, with the advantage of minimal sample preparation and compatibility with wet or dry samples, offering information about their morphology and size [77]. In previous studies, AFM was used to characterize AgNPs produced from *Tribulus terrestris* extract, revealing an average size of 60 nm [78], from four different *Aspergillus* species, with a highly variable morphology and spherical shape [79] or from Bacillus with sizes between 12 and 20 nm, and also a spherical shape [80]. The limitations of this technique fall on the possible interaction of the tip with the sample, which can affect the results and the long analysis time.

### Dynamic light scattering (DLS)

It measures the hydrodynamic diameter of NPs in suspension, including their shell, and provides information on size distribution and zeta potential, which is related to colloidal stability [81]. For instance, DLS allowed for the determination of the diameters and zero potential values of silver and gold NPs synthesized with *Echinophora platyloba* extract (diameters: 24 and 139 nm, zeta potentials: −12 to −48 mV, respectively) [82], or of AgNPs synthesized with *Annona diversifolia* extract [83] (average diameter: 34 nm, zeta potential: −20.2 mV) and *Melia azedarach* extract (average diameter: 32 nm, zeta potential: −13.1 mV) [84]. The principal limitations of the technique are the overestimation of the real sizes, especially when the NPs are dispersed in size and covered by biomolecules, and the sensitivity to aggregates, impurities, or media conditions such as viscosity and salt content.

### X-ray-based techniques

X-ray-based techniques, including X-ray fluorescence (XRF) and transmission X-ray microscopy (TXM), enable elemental analysis and 3D imaging of NPs within biological matrices [85]. These methods were used to image AuNPs produced by *Saccharomyces cerevisiae* [85]. However, their use in bioNPs characterization is not widely spread due to their limited spatial resolution and analysis restriction to dispersed or trace-concentrated NPs, and to the expensive specialized instrumentation and complex sample preparation, respectively. On the other side, X-ray diffraction (XRD) provides crystallographic and composition/purity information, as shown in the characterization of SeNPs from *Pseudomonas aeruginosa* JS-11, revealing a face-centred cubic structure with an average size of 21 nm [86] or in the characterization of AgNPs from *Rhizoctonia solani* and *Cladosporium cladosporioides*, revealing a high-purity crystalline structure with sizes ranging between 80 and 100 nm [87]. The main problem of its application to bioNPs is that the samples should be relatively pure, crystalline, and dry, which requires a cleaning protocol for the complex biological matrix, since the presence of commonly formed organic covers can underestimate the results.

### Energy-dispersive X-ray spectroscopy (EDX)

Often coupled with TEM or SEM, it is based on the emission of energy in the form of X-rays that are characteristic of the elements present in the sample and provide information on its composition. EDX was used, for instance, to confirm the composition of zinc oxide NPs as 52% zinc and 42% oxygen synthesized from various seaweeds (green *Caulerpa peltata*, red *Hypnea Valencia*, and brown *Sargassum myriocystum*) [88] to prove the formation of pure iron oxide NPs (77.08% Fe and 22.97% O) from an *Eichhornia crassipes* leaf extract [89] or to know the high-purity composition of Ag and Au NPs synthesized in the presence of *Crataegus monogyna* leaf extract [90].

### Single-particle inductively coupled plasma mass spectrometry (spICP-MS)

spICP-MS analyses diluted NP suspensions by detecting individual particles as discrete signal peaks. This accurate analysis provides information on the size (if the shape, elemental composition, and density of the NPs are known) and the particle number concentration [91]. This allowed following the transformation of spherical TeNPs incubated in *Staphylococcus aureus* and *Escherichia coli* to nanorods [92], copper bioNPs from *Streptomyces coelicolor* [93] or the presence of nanoparticulate forms of polyphosphate in the same bacterial species [27, 36]. The main drawbacks of spICP-MS are the achievable size detection limits (usually > 10 nm in diameter) and the need to complement the data with image and structural techniques to validate the results.

### Hyphenated techniques coupled with ICP-MS

Flow field fractionation (FFF) is a non-chromatographic technique that separates NPs by size based on their different diffusion rates in a flow channel. The most common form, asymmetric flow field-flow fractionation (AF4), allows efficient separation of NPs between 1 and 500 nm without a stationary phase [94]. The combination of AF4 and ICP-MS enables elemental analysis of separated NPs, also defining size distribution and aggregation state [95]. This type of technique has been applied for the qualitative and quantitative analysis of various synthetic NPs, such as AgNPs and AuNPs, in human urine, blood, and serum [96]. However, despite its applicability, there are few examples of its application to bioNPs. The main reasons could be related to its high operational complexity, low performance in samples rich in organic matter, adsorption losses on the membrane, the need for specific standards, and high analysis costs. One example in the analysis of bioNPs is the identification and characterization of SeNPs extracted from lactic acid bacteria, with results confirmed by DLS [63]. Alternatively, high-performance liquid chromatography (HPLC) coupled to ICP-MS showed its general applicability to separate NPs by size (< 40 nm in diameter). In the case of bioNPs, the analysis of selenized yeast demonstrated the presence of small SeNPs (approx. 4–6 nm in diameter) not detectable by spICP-MS [36]. Recently, this methodology also revealed the presence of Fe- and Al-containing NPs in farmed insects [97].

According to what has been described in this section, there is no technique capable of offering a complete characterization of the nanoparticle core. Due to this, the use of complementary techniques that allow obtaining information about the size or shape, such as microscopy, the composition, such as X-ray-based techniques, or the concentration, such as mass spectrometry, is mandatory, since these will be decisive in the applications to which these nanoparticles are suitable.

## Chemical characterization techniques for the surface properties of the nanoparticles

The surface chemistry of NPs is critical for their stability, biological interactions, and functionality, especially for biogenic nanostructures, where a biomolecular capping layer greatly influences their biological identity and uptake [98]. Understanding the surface composition, including the PC, is essential before any application (Fig. 6, Table 2).

### UV–vis spectrophotometry

UV–Vis spectrophotometry measures the absorption of light in the 170–780 nm range to generate characteristic spectra [99]. For metallic NPs, surface plasmon resonance (SPR) is highly sensitive to the local environment—adsorption of biomolecules causes a red shift in the SPR peak, indicating dielectric layer formation [100]. This technique has been widely used to study PC formation and NP aggregation [101, 102]. In the case of bioNPs, only one report has used UV–vis to distinguish chemically synthesized AgNPs (SPR at 414 nm) from biogenic AgNPs produced by *Macrophomina phaseolina* (SPR at 420 nm), also demonstrating greater long-term stability for the bioNPs [103]. However, there are various examples of synthetic NPs. For instance, Dominguez-Medina et al*.* performed UV–Vis measurements of citrate-stabilized AuNPs in the presence of bovine serum albumin (BSA), reporting a red shift of the SPR peak from 534 to 537 nm [104]. This spectral change directly indicated BSA adsorption and conformational changes in the corona layer. Similarly, in 2022, Bondžić et al*.* employed UV–Vis spectroscopy to monitor the interaction between L-methionine-capped AgNPs and BSA, observing a red shift of the SPR peak from 424 to 427 nm as BSA concentration increased [105]. These studies demonstrate that UV–Vis spectrophotometry is a valuable tool for easily monitoring the formation of the PC. However, despite its simplicity, it offers only indirect evidence of corona formation and lacks the specificity to identify even the nature of the adsorbed molecules or their conformational state. Therefore, it can only be used as a preliminary or together with complementary techniques that provide more detailed structural or proteomic information.

### Fourier transform infrared spectroscopy (FTIR)

FTIR operates by measuring absorption in the mid-infrared range (4000–400 cm⁻^1^) to identify chemical bonds and functional groups, providing insight into molecular structures and interactions. This technique is particularly valuable for characterizing biomolecules present on the surface of bioNPs, detecting protein conformations within the PC, and monitoring the binding interactions between the PC and NPs [106]. A notable example is provided by Kamnev et al*.* [107], who used FTIR analysis to study the effects of sample pre-treatment, such as washing, on the biomolecular capping layer of biogenically generated SeNPs produced by *Azospirillum brasilense* bacterial strain. Their findings demonstrated that with increasing washing steps, the FTIR bands at ~ 564 and ~ 1412 cm^−1^—attributed to the antisymmetric and symmetric stretching vibrations of ionized carboxylate residues—disappeared. This result indicated that carboxylate-containing species, such as carboxypolysaccharides, were only weakly bound to the NP surface and could be removed by washing. Additionally, the identification of amide I and II bands (around 1650 and 1540 cm^−1^) confirmed the presence of proteins, while ester stretching vibration bands (near 1740 cm^−1^), indicated the presence of lipids on the NP surface. Another relevant example is provided by Kar et al*.* [108] who employed FTIR to characterize AuNPs biosynthesized by the phytopathogenic fungus *Nigrospora oryzae*. The presence of amide I (~ 1652 cm⁻^1^) and amide II (~ 1543 cm⁻^1^) bands in the spectra confirmed that proteins were involved in the capping and stabilization of the biogenic AuNPs. Valuable insights can also be drawn from studies using synthetically produced nanomaterials. For instance, Podila et al*.* used FTIR to monitor conformational changes in BSA adsorbed onto AgNPs with different surface coatings [109]. By analyzing the amide I band, they quantified shifts in protein secondary structure, particularly the transition from α-helices (~ 1650 cm⁻^1^) to β-sheet structures (~ 1630 cm⁻^1^). This work highlights the sensitivity of FTIR in detecting PC conformational changes linked, in this case, to NP surface chemistry. However, FTIR also presents several limitations in this context. The technique lacks the specificity to distinguish between and identify individual proteins within complex coronas. Moreover, overlapping absorption bands can complicate spectral interpretation.

### Surface-enhanced Raman spectroscopy (SERS)

SERS builds upon conventional Raman spectroscopy, which relies on the inelastic scattering of monochromatic laser light to probe molecular systems. The resulting vibrational spectrum serves as a molecular fingerprint, offering detailed chemical and structural information about the sample [110]. However, Raman spectroscopy is limited by its low sensitivity, as only a minor fraction of light undergoes inelastic scattering.

SERS addresses the limitation by significantly amplifying the Raman signal of molecules. This enhancement is primarily achieved using metallic nanostructures, which act as probes and locally intensify the incident electromagnetic field via SPR effects. Additionally, chemical interactions between the metal surface and the target molecules contribute to further signal amplification [111].

Through these mechanisms, SERS enables the investigation of PC conformations and side-chain environments based on their vibrational characteristics. Notably, there remains a gap in the scientific literature regarding the application of SERS to study PC on biogenically generated NPs, and there are few applications regarding synthetic NPs. An illustrative example is the study by Szekeres et al. [112], who applied SERS (in combination with 1D PAGE with following LC–MS/MS) to investigate the fragmentation of PC components in AuNPs internalized by live J774 cells. By comparing spectra from intact cells and cytoplasmic extracts, they observed specific spectral changes associated with protein denaturation and enzymatic digestion occurring within endolysosomal compartments. In parallel, control experiments using trypsin-digested BSA revealed a loss of amide II and III bands, features that were indicative of corona protein fragmentation upon cellular processing. Such results demonstrate the ability of SERS to monitor conformational alterations in PCs under biologically relevant conditions. Nonetheless, SERS does not provide information about the overall composition of the PC and applies only to metallic NPs [113]. Due to these limitations, SERS is often used in conjunction with complementary techniques such as FTIR to achieve a more comprehensive characterization [114].

### Liquid chromatography coupled to tandem mass spectrometry (LC–MS/MS)

This technique is one of the most powerful technologies for proteomic analysis, enabling both qualitative and quantitative characterization of the PC surrounding NPs [100]. Although this strategy has been widely employed to characterize the composition of PCs formed around NPs when exposed to biological media, its use in the characterization of PCs of bioNPs has been little described yet. For instance, Spagnoletti et al*.* utilized LC–MS/MS to identify the capping proteins associated with biogenically synthesized AgNPs produced by cell-free filtrates of *Macrophomina phaseolina.* After extraction and purification, peptides were analyzed using a nanoLC instrument coupled to an ESI source and a tandem quadrupole-Orbitrap mass analyzer. The study identified 46 proteins from the fungal proteome, which were further classified using the UniProt database [103]. In another example, Nie et al*.* identified vacuolar protease (PEP4p) as the predominant protein coating intracellularly generated SeNPs in *Saccharomyces boulardii.* This identification was achieved by isolating the most abundant protein band using SDS-PAGE, digesting it, and analyzing the resulting peptides with LC–MS/MS [115]. There are also studies involving intracellularly formed PC but in chemically synthesized NPs. For example, Qin et al*.* investigated the intracellular PC of chemically synthesized AuNPs after transcytosis across Caco-2 cells, identifying over 200 associated proteins, and revealing complex intracellular interactions [116]. LC–MS/MS is a powerful tool for PC characterization; however, it still presents certain limitations, particularly in the case of bioNPs. These limitations are mainly associated with the sample preparation steps, which may lead to the loss of weakly bound proteins from the soft corona and introduce variability in the results.

### Optical microscopy

More recently, advancements in optical microscopy have enabled more precise and dynamic analysis of NP–PC interactions in complex biological environments. Two notable techniques, Confocal Laser Scanning Microscopy combined with Microfluidics (CLSM-MF) and Hyperspectral-Enhanced Dark-Field Microscopy (HEDFM), offer powerful and complementary approaches for studying the formation, behaviour, and identification of NPs and their associated biomolecular layers in situ. CLSM-MF offers a powerful approach for studying in situ kinetic formation of the entire PC under controlled flow conditions. Weiss et al*.* pioneered this technique to provide a clear distinction between the “hard” and “soft” PC, enabling real-time observation of protein adsorption onto NPs [117]. The integration of microfluidic systems allows for precise control over key experimental parameters, such as channel dimensions and flow rates, resulting in highly reproducible and standardized experimental setups [118]. This level of control enhances the reliability of data and supports the standardization of both experimental procedures and data reporting. Using the MF-CLSM configuration, researchers can conduct both static and dynamic experiments, as well as assess the stability of the PC. This technique has revealed the existence of three distinct adsorption regimes, each corresponding to the formation of unique phases during PC development. It is important to note, however, that this methodology has primarily been applied to monitor the formation of the PC rather than to characterize pre-formed coronas on bioNPs. Therefore, adapting this approach for the study of already established PC on bioNPs would require further methodological development and optimization. HEDFM, which integrates dark-field microscopy with hyperspectral imaging (HIS) [119], is an innovative optical technique with great potential in biological research, allowing the identification and quantitative analysis of specific components within complex biological environments. However, this method often results in lower optical resolution, reduced signal-to-noise ratios, and an out-of-focus appearance due to the combined effects of reflected and transmitted light. In contrast, dark-field microscopy captures only the light reflected or elastically scattered by the sample, effectively eliminating background noise and providing high-contrast images [120]. This technique is particularly advantageous for observing low-contrast specimens that are typically invisible under conventional bright field conditions. The addition of hyperspectral imaging further enhances this technique by allowing the collection of spectral information from each pixel in the image, thus enabling detailed analysis of the composition and behaviour of NPs in situ*.* For example, Shannahan et al*.* [121] employed hyperspectral dark-field microscopy to study AgNPs in phagolysosomal fluid, focusing on changes in the spectral properties of internalized NPs. Their findings revealed that the shifts in the light-scattering spectra of NPs with a PC were significantly greater than those observed for AgNPs alone, highlighting the impact of the biological environment and PC formation on NP behaviour.

Similar to the strategies for the characterization of the particle core, none of the above-mentioned analytical methods can offer a complete picture of the surface composition. For this aim, the complementary use of diverse techniques is still lacking in many case studies.

## Conclusions and future perspectives

The biogenic synthesis of NPs, using plants, fungi, bacteria, and other biological entities represents a promising and sustainable alternative to conventional chemical methods. This green approach avoids harmful reagents and harsh reaction conditions. It often produces NPs naturally coated with biomolecules that form a PC, which may equip them with unique biological functionalities, boosting antitumoral and anti-inflammatory effects. However, a significant gap in current research is the lack of a clear understanding of the dynamic and complex in situ formation of NPs by living organisms. Most studies have focused on extract-mediated “pseudo-green” approaches, which do not fully capture the real-time synthesis process. Without a clear understanding of these distinct pathways, the mechanisms of corona formation, NP evolution, and their ultimate biological behaviour remain insufficiently elucidated.

To unravel these complexities, advanced bioanalytical techniques are essential, each presenting distinct advantages and limitations. While traditional methods like EM, including TEM and SEM modes, offer unmatched spatial resolution and detailed visualization of NP morphology, size, and intracellular localization, TEM provides high-resolution and tomographic imaging valuable for studying subcellular NP interactions. However, it requires rigorous sample preparation and vacuum conditions that may introduce artifacts or alter the native state of biological samples. SEM allows broader surface examinations with less stringent sample thickness requirements but provides somewhat lower resolution. Both methods are time-consuming and typically analyze limited sample volumes, which can limit throughput and quantitative analysis. Complementing EM, AFM delivers high-resolution surface topography under ambient or liquid conditions without vacuum, thus better preserving biological structures. Nonetheless, AFM is low throughput and sensitive to tip-sample interactions, which reduces its suitability for statistical analyses. DLS enables rapid evaluation of NP hydrodynamic size and colloidal stability in suspension, but its accuracy declines when samples feature broad size distributions or irregular particle shapes. Alternatively, spectroscopic techniques offer molecular-level insights: UV–Vis spectrophotometry is a simple and fast method to monitor NP SPR and aggregation, though it lacks molecular specificity. Conversely, FTIR effectively identifies functional groups and molecular interactions on NP surfaces but may struggle with overlapping signals in complex biological matrices. SERS improves vibrational fingerprinting sensitivity, especially for metallic NPs, facilitating detailed protein conformation studies. However, its reliance on metallic substrates restricts its application scope and it does not provide comprehensive compositional information. Finally, LC–MS/MS excels at detailed qualitative and quantitative proteomic profiling of the PC, revealing the identity and abundance of surface-bound biomolecules with high sensitivity. Yet, the technique is labor-intensive, requires specialized equipment and expertise, and may not be routinely accessible.

In this context, mass cytometry (CyTOF) emerges as a particularly powerful bioanalytical approach, as it can combine the multiplexing abilities of flow cytometry with the elemental and molecular selectivity of time-of-flight mass spectrometry to provide rapid, sensitive, single-particle analysis of both metal content and PC composition within complex biological fluids. This could make CyTOF especially valuable for characterizing the biological identity of bioNPs in situ, a critical step towards understanding their function and fate.

Looking ahead, the application scope and prospects for analytical science in this field are immense. Key challenges lie in developing experimental platforms that allow for real-time, in vivo, or native-condition studies of bioNP formation and corona dynamics, instead of relying solely on the analysis of purified particles. Even the isolation and extraction of biogenically produced NPs are still a challenge and require, in the future, harmonized and reliable protocols. We believe that integrative analytical approaches that combine the strengths of techniques like CyTOF with imaging and spectroscopic methods are essential to generate the multidimensional datasets needed to truly correlate NP structure, surface chemistry, and biological behavior. Moreover, it remains imperative to explore how varying biological environments influence NP synthesis, corona formation, toxicity, and therapeutic efficacy. Addressing the challenges of reproducibility and scaling up green synthesis methods is also vital for transitioning bioNP applications from research to clinical and industrial settings.

In conclusion, while green synthesis of NPs offers a sustainable and biologically relevant path for NP production, the lack of thorough investigations into real-time biogenic formation represents a significant gap. A comprehensive bioanalytical approach that benefits from the strengths and compensates for the limitations of each technique will be critical to deepen our understanding and enable the safe and effective use of bioNPs.

## Data Availability

Not applicable.
